# Pyoperitoneum revealing a spontaneous renal forniceal rupture due to ureteropelvic junction syndrome: About a rare case report

**DOI:** 10.1016/j.eucr.2022.102231

**Published:** 2022-09-21

**Authors:** Anouar Elmoudane, Ibrahim Boukhannous, Anass Elalaoui, Wassim Alaoui Mhammedi, Mohamed Mokhtari, Ali Barki

**Affiliations:** a Department of Urology, Mohamed VI University Hospital Center, Mohamed I University, Oujda, Morocco; b Department Head of Urology, Mohamed VI University Hospital Center, Mohamed I University, Oujda, 62000, Morocco

**Keywords:** Ureteropyelic junction syndrome, Forniceal rupture, Peritonitis, Pyonephrosis

## Abstract

Extravasation of urine following forniceal rupture of a pelviureteric junction is a rare complication; the existence of pyonephrosis can result to retroperitoneal abscess but its fistulization into peritoneal cavity is exceptional. We report a case of a 22-year-old men who presented a clinical aspects of peritonitis, abdominal CT scan findings suggested retroperitoneal peritonitis by rupture of the fornix.

This case emphasizes an unusual presentation of pyonephrosis with peritonitis and pyoperitoneum caused by a ureteropelvic junction syndrome.

## 1 Introduction

Renal forniceal rupture is a rare evolution of hydronephrosis secondary to an obstruction of the urinary tract. The majority of cases are attributed to ureteral calculi but other aetiologies have been described in the literature such as extrinsic ureteral compression, pregnancy, posterior urethral valve, I.V fluid administration and in exceptional cases due to pyelo-ureteric junction syndrome.^1^ Hydronephrosis may be complicated by infection and lead to pyonephrosis extending into the perirenal space and the psoas muscle. Intraperitoneal fistulization occurs very rarely and may lead to a presentation of generalized peritonitis.

## 2 Case presentation

A 22 years old male patient without medical history was admitted to the emergency room for diffuse abdominal pain starting at the right lumbar region for one month. At the clinical examination, the patient was conscious, hemodynamic and respiratory stable, febrile at 39 C°, with diffuse abdominal contracture.

**Fig. 1:**
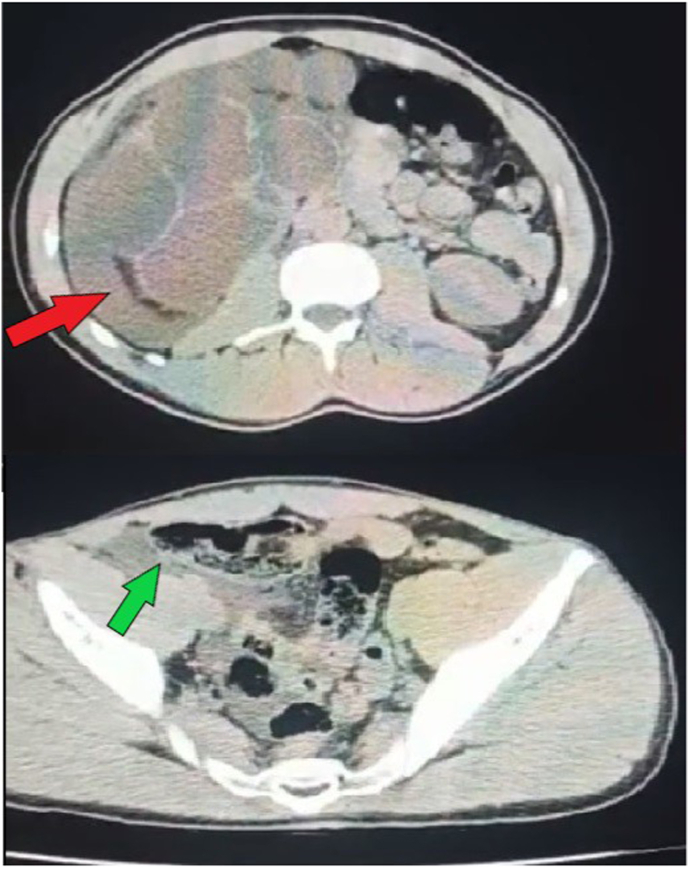
Axial section of a CT abdomen showing perinephric collection (red arrow) and intraperitoneal collection (green arrow). (For interpretation of the references to colour in this figure legend, the reader is referred to the Web version of this article.)

**Fig. 2:**
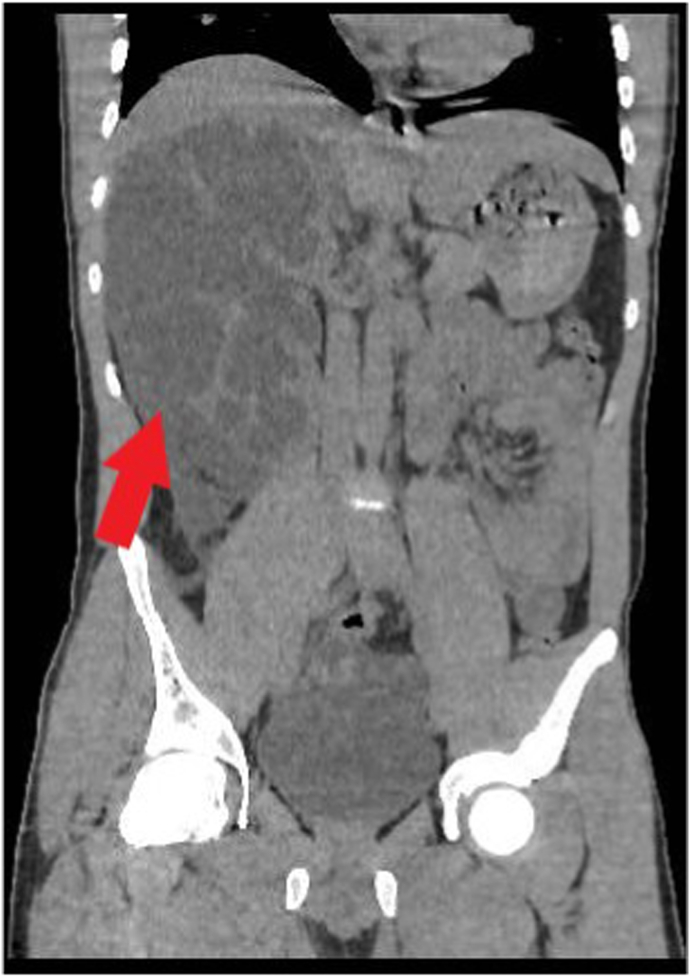
Coronal section of CT abdomen showing perinephric collection (red arrow). (For interpretation of the references to colour in this figure legend, the reader is referred to the Web version of this article.)

An injected abdominopelvic scan revealed (Fig. 1; Fig. 2) a significant right pyelo-ureteral dilatation laminating the renal parenchyma without visible obstacle with a thin ureter, associated with Peri-renal effusion and late of contrast material extravasation to the retroperitoneal and intraperitoneal regions, suggesting retroperitoneal peritonitis by rupture of the fornix, thus a uretero-pelvic junction obstruction (UPJO) might be the possible cause of pyonephrosis.

Biological assessment objectified an infectious syndrome with a high-level CRP at 319 mg/l, and a high level of white blood cells at 17200/mm3. A hemoglobin level at 15 g/dl, platelets at 257000/mm3. An acute renal failure with creatinine level at 17 mg/L, urea at 0.4 g/L, with normal blood ionogram. The ECBU objectified a leucocyturia at 920000 and hematuria at 120000. Urine culture found a pseudomonas earoginosa.

The patient underwent initially a right nephrostomy who has drained a 2L of pus to prevent septic shock in patients, he received I.V fluids and I.V antibiotics; in front of the ruined kidney, a left nephrectomy with a good peritoneal lavage and positioning of a drain was performed. The patient recovered well with resolution of sepsis and discharged on 6th postoperative day after removal of abdominal drains.

## 3 Discussion

Pyoperitoneum and peritonitis are commonly due to an intraperitoneal pathology generally of digestive origin (gallstone, pancreatitis …). The peritoneal irritation caused by a pyelonephritis can lead to a peritonitis and the evolution is usually benign. However, a pyonephrosis rupture leading to intraperitoneal collection is rarely reported in the literature.

Spontaneous Renal fornical rupture is a rare complication of obstructive uropathy due to hydronephrosis and overpressure exerted on the renal parenchyma. Many etiologies can be involved, especially ureteral calculi, tumors, and in exceptional cases ureteropelvic junction syndrome.^1^ In case of rupture of the renal fornix with pyonephrosis, the pus is normally limited to the retroperitoneal region, as there is no anatomical communication with the intraperitoneal or pelvic region.

The clinical presentation is not always specific, usually, pyonephrosis is characterized by a clinical sign of lumbar pain with fever. A rupture of the fornix due to a junction syndrome that results in peritonitis is a very rare case. The CT scan confirms the diagnosis by showing in the excretory phase with contrast material extravasation to the retroperitoneal and intraperitoneal regions.^2^

The treatment of a ruptured pyonephrosis with antibiotics alone is not effective and must be combined with a drainage using either a nephrostomy or ureteral stent to prevent septic shock in patients.

Shifti and beleke reported a case of ruptured pyonephrosis causing a peritonitis in a patient with a pelvic-ureteral junction with a damaged kidney managed by nephrectomy.^3^ Hendaoui in 1999 reported a case of intraperitoneal extension in patient with non-functioning kidney due to urolithiasis.^2^ A case of spontaneous rupture of pyonephrosis leading to pyoperitoneum even in absence of obstruction of urinary tract.^4^ A recent case was reported of a patient who presented spontaneous rupture of pyonephrosis into anterior abdominal wall.^5^

In our case, the patient was treated in the emergency department with bi-antibiotic therapy and percutaneous drainage to prevent septic shock. Afterwards, he underwent a right nephrectomy considering that the kidney had been ruined; the postoperative follow-up was favorable.

## 4 Conclusion

Spontaneous rupture of the renal fornix is not common, the transmission of pyonephrosis to the intraperitoneal region is possible and should be recognized by the physician for more efficient management of the patient and to prevent complications.

## Declaration of competing interest

None of the contributing authors have any conflict of interest, including specific financial interests or relationships and affiliations relevant to the subject matter or materials discussed in the manuscript.
